# Impact of Wood Smoke Exposure on Aortic Valve Mineralization: Microvesicles as Mineral Conveyors in Patients with Coronary Stenosis

**DOI:** 10.3390/jcm14010146

**Published:** 2024-12-30

**Authors:** Mirthala Flores-García, Carlos Linares-López, Valentin Herrera-Alarcón, Elizabeth Soria-Castro, Marco Antonio Peña-Duque, Adolfo Arellano-Martínez, Guillermo Cardoso-Saldaña, Benny Giovanni Cazarín-Santos, Esbeidy García-Flores, Eduardo Angles-Cano, Aurora de la Peña-Díaz

**Affiliations:** 1 Molecular Biology Department, Surgery and Cardiovascular Biomedicine, National Institute of Cardiology Ignacio Chávez, Juan Badiano 1, Tlalpan, Mexico City 14080, Mexico; mirthala.flores@cardiologia.org.mx (M.F.-G.); ulisesmed98@gmail.com (V.H.-A.); elizabethsoria824@gmail.com (E.S.-C.); cargui@cardiologia.org.mx (G.C.-S.); benny.cazarin@facmed.unam.mx (B.G.C.-S.); esbeidy.garciaflores@gmail.com (E.G.-F.); 2 Institute of Geophysics, National Autonomous University of Mexico, Circuito Escolar, Ciudad Universitaria, Coyoacán, Mexico City 04510, Mexico; linaresc@geofisica.unam.mx; 3 Cardiology Unit, Médica Sur Hospital, Puente de Piedra 150, Toriello Guerra, Tlalpan, Mexico City 14050, Mexico; marcopduque@gmail.com; 4 Regional Hospital of ISSSTE Dr. Valentín Gómez Farias, Prolongación Américas 203, Zapopan 45100, Jalisco, Mexico; arm_6215@hotmail.com; 5 Pharmacology Department, Faculty of Medicine, National Autonomous University of Mexico, Circuito Escolar, Ciudad Universitaria, Coyoacán, Mexico City 04510, Mexico; 6 INSERM UMR_S-1140 & UMR_S-1144, Innovation Diagnostique et Thérapeutique en Pathologies Cérébrovasculaires et Thrombotiques, Faculté de Pharmacie de Paris, Université Paris Cité, 75006 Paris, France

**Keywords:** environmental factor, wood smoke, valve calcification, microvesicles

## Abstract

**Background:** Aortic valve calcification results from degenerative processes associated with several pathologies. These processes are influenced by age, chronic inflammation, and high concentrations of phosphate ions in the plasma, which contribute to induce mineralization in the aortic valve and deterioration of cardiovascular health. Environmental factors, such as wood smoke that emits harmful and carcinogenic pollutants, carbon monoxide (CO), and nitrogen oxide (NO_x_), as well as other reactive compounds may also be implicated. The purpose of this research was to study the impact of wood smoke on specific aortic valve characteristics, including lesion size and percentage of mineralization, in patients with aortic valve stenosis (AS). **Methods:** This observational study included 65 patients who underwent primary valve replacement surgery at the National Institute of Cardiology, 11 of whom were exposed to wood smoke. For each patient, approximately 0.5 cm of aortic valve tissue was collected along with a blood sample anticoagulated with sodium citrate. The valves were analyzed using scanning electron microscopy coupled with energy-dispersive X-ray spectroscopy (SEM–EDS). Since extracellular microvesicles (MVs) may induce epigenetic changes in target cells by transferring their cargo, we also analyzed their mineral content. **Results:** Individuals exposed to wood smoke exhibit more extensive lesion (835 µm^2^) characteristics compared to those with no exposure (407.5 µm^2^). Interestingly, FESEM images of MVs showed the presence of minerals on their surface, thus providing evidence on their possible role in the pathophysiology of mineralization. **Conclusions:** Our study uniquely demonstrates imaging-based evidence of structural damage and mineralization in aortic valve tissue, with chronic wood smoke exposure emerging as a significant causative factor.

## 1. Introduction

Aortic valve calcification (VC) is the result of degenerative, congenital, or rheumatic processes; regardless of the origin, different associated mechanisms can intervene in all of them, such as chronic inflammation, high plasma concentration of phosphate ions, and the presence of environmental particulate matter (PM) released from wood burning, mainly 2.5 µm (PM2.5), all of which are associated with the deterioration of cardiovascular health [1].

There are several forms of environmental pollution, for example, that are caused by transportation combustion gases, cigarette smoke, and industrial smoke; however, in wood smoke, the presence of suspended particles of small PM is linked to greater morbidity and mortality [2].

PM2.5 is a measure that is taken as a reference for small particles in environmental pollution that, both in the short- and long-term, increases cardiovascular risk and reduces life expectancy. Wood smoke emits harmful and carcinogenic pollutants. CO and NOx as well as other reactive compounds produced by the combustion of biomass cause, in exposed people, not only cardiovascular diseases but also short- and medium-term toxic effects that produce oxidative lesions, cancer, teratogenesis, and allergenic and mutagenic effects [2,3]. The final consequence, regardless of the process involved, is the loss of the mechanical function of the valve. Its incidence has increased markedly in parallel with the growth in life expectancy of the population. To preserve adequate mechanical function of the aortic valve, its microarchitecture must be maintained as well as the function of the different areas that make it up, mainly the internal or endothelial area, which is in contact with the blood and the area where interstitial cells are present.

Upon exposure to biochemical mediators, including environmental factors, resident valve endothelial and interstitial cells may undergo endothelial-to-mesenchymal transition or osteoblast-like differentiation, respectively. They release extracellular vesicle cells that, with the osteogenic activity, serve as a nidus for microcalcifications. Each of these mechanisms has been shown to contribute to aortic valve calcification. Microcalcifications colocalize with lipids in the presence of extracellular MVs, which induce crystalline formation of hydroxyapatite (calcium orthophosphate), and are nucleation sites for greater mineral deposition [4].

The microenvironment is a factor that has an impact on modulating the content of MVs, modifying their content and, therefore, their biological activity and interaction with different cellular targets through the transport of molecules, such as growth factors, lipids, or miRNAs, among others [5].

The association between the development of valvular disease and environmental factors suggests that epigenetic control also participates in both its genesis and development [6,7]. In fact, the relationship between exposure to smoke from forest fires and death from cardiovascular causes [8] is consistent in most epidemiological studies. Exposure to indoor fuel wood smoke has been shown, in some studies, to cause carotid thickening, increased blood pressure, and endothelial dysfunction [9]. The results of the MESA [10] study show a clear trend between pollution from industrial activity and fuels and mitral calcification in individuals without cardiovascular disease. Study strategies to elucidate the molecular mechanisms involved in valve calcification include in vivo animal models and in vitro culture cell models.

In humans, epidemiological studies use non-invasive strategies to research vascular or valvular calcification, including detection by ultrasound and echocardiography, or more precise definition by computed tomography and positron emission tomography. A novel study strategy included the evaluation of valve material ex vivo that combined scanning electron microscopy with energy-dispersive X-ray spectroscopy (SEM–EDS). This alternative study showed the precise elemental composition of the valves and revealed the characteristics of the extracellular vesicles, MVs, that initiate the process [11].

The objective of this study was to identify the presence of minerals on MVs in individuals exposed to wood smoke as a proof of concept and to elucidate the qualitative and quantitative differences in the minerals present in the aortic valve.

## 2. Materials and Methods

A cross-sectional, analytical, descriptive study was carried out in which we analyzed the aortic valves of adult patients, both sexes, who underwent, regardless of the cause, primary valve replacement surgery at the National Institute of Cardiology Ignacio Chávez, City of Mexico (INC). The decision to proceed with aortic valve replacement (AVR) depends on the severity of aortic valve stenosis (AS), the presence of symptoms, and left ventricular function. The severity of AS is typically assessed using echocardiographic measurements, including the aortic valve area (AVA), mean pressure gradient (MPG), and peak aortic jet velocity. The decision to proceed with AVR was taken depending on the presence of symptoms and left ventricular function. According to the 2020 ACC/AHA [12] guideline, the aortic valves selected for this study were from patients who had a degree of severity classified as symptomatic severe AS (AVA < 1.0 cm^2^, MPG > 4.0 m/s: peak aortic velocity 4.0 m/s). AVR was, therefore, indicated for patients with severe AS who exhibit symptoms such as angina, syncope, or heart failure.

A total of 65 patients were included, by convenience, between 2014 and 2016. Eleven of the patients were exposed to wood smoke due to work or indoor conditions, and seven of them had at least thirty years of exposure. Patients with a diagnosis of cancer and a history of acute coronary ischemic syndrome, thyroid disease, or acute liver or kidney disease were not included. This protocol was carried out following the ethical principles of the Declaration of Helsinki. The patients gave their written informed consent, and the protocol was approved by the Medicine Faculty—UNAM Ethical and Research Committees with number register FIMICOR 047/2014.

From each patient, ± 0.5 cm of tissue (aortic valve) was obtained during valve replacement surgery and immediately preserved in 10% paraformaldehyde (MERCK, Burlington, Massachusetts, USA) until subsequent analysis. These samples were dried at room temperature for 24 to 48 h, placed on coverslips, covered with graphite, and studied with a SEM–EDS Model JXA8900-R (JEOL, Peabody, MA, USA) at an acceleration voltage of 20 kilo electrovolts, an acquisition time of 30 at 60 s, and a current of 20 nanoamperes. Digital images were obtained with a resolution of 1024 × 1024 pixels, and they were transformed into images with shades of gray.

To avoid bias, the images had the same resolution and were processed by placing them in the Image-Pro Plus 7.0 program. With the same orientation, an area of the same size was chosen in the same coordinates, and the surface of the mineralized area of each sample was obtained. The image intensity is expressed as a percentage of mineralization, and the different minerals present in the tissue are obtained from the scanning electron microscope analysis as a percentage.

The image of the minerals present in the MVs was obtained from the plasma from three patients exposed to wood smoke. The MVs were obtained as described in Valente-Acosta et al. [13] with modifications. Briefly, MVs were isolated from a blood sample anticoagulated with sodium citrate (0.109 M). The sample was centrifuged at 1500× *g* for 15 min to remove cells and cellular debris. The platelet-poor plasma (PPP) was further submitted twice to centrifugation at 13,000× *g* for 2 min to obtain platelet-free plasma (PFP). The MVs were then separated from 500 µL of PFP by centrifugation twice at 20,000× *g* for 90 min at 4 °C and suspended in 100 µL of deionized water. The amount of MVs was quantitated by measuring protein concentration at A_280nm_ in MV lysates produced by thermic shock (5 cycles at 37 °C and −180 °C) using a NanoDrop^®^ ND-1000 spectrophotometer (Thermo Fisher Scientific, Waltham, MA, USA). The concentration is expressed in mg/mL of protein.

For the electron microscopy studies, 30 µL of the suspension containing the MVs from three patients at a concentration of 0.150 mg/mL of protein was placed on a nickel grid coated with carbon and subsequently in a desiccator (to eliminate all moisture). The measurement was carried out on a field emission scanning electron microscope (FESEM) Model JSM-7401F (JEOL, MA, USA) instrument. The various areas of each sample were evaluated at 5 and 10 KeV. The potential that excited all the elements in the sample was 10 KeV, achieving this energy to stimulate both the K and L levels of the searched elements. The spectrum was obtained at 10 KeV for microanalysis for 300 s. The evaluation area was 200 nm.

### Statistical Analysis

Variables are shown as the median (interquartile range) or *n* (%) due to data type and distribution. * *p* < 0.05 was considered statistically significant. Shapiro–Wilks and Mann–Whitney U or chi-square tests were performed with the SPSS v23.0 program (SPSS Inc., Chicago, IL, USA).

## 3. Results

The demographic and clinical characteristics are presented in Table 1 and Table 2, which were obtained from clinical records. 

### 3.1. Mineralization Analysis of Aortic Valve

**Figure 1:**
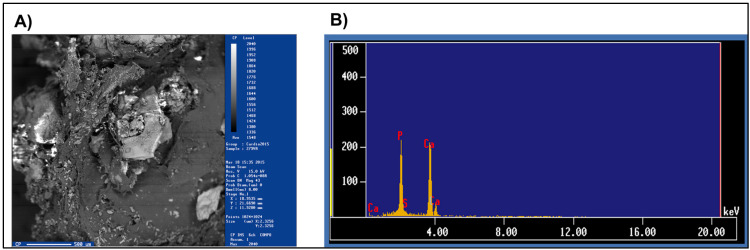
A representative image of the results of a studied aortic valve. (**A**) Image of a mineralized aortic valve; (**B**) histogram of the elements present in the tissue. A high-resolution, three-dimensional digital image is created by a beam of backscattered electrons that impinge on the surface of the valve. In addition to the structural information (morphology and topology) of the sample, this technique allows for the determination of its elemental composition due to data generated by X-rays. The minerals present in the sample are observed as white/silvery spots with an amorphous shape, which contrast with the tissue (darker background) due to the high emission intensity of backscattered electrons emitted by minerals. To avoid bias, the images had the same resolution and were processed by placing them in the Image-Pro Plus 7.0 program. With the same orientation, an area of the same size was chosen in the same coordinates, and the surface of the mineralized area of each sample was obtained. The image intensity is expressed as a percentage of mineralization, and the different minerals present in the tissue are obtained from the scanning electron microscope analysis as a percentage.

**Figure 2:**
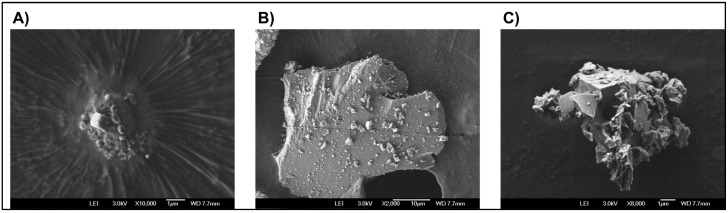
Representative images of MVs present in the plasma from three patients exposed to wood smoke. The three images (**A**–**C**) were obtained by a field emission scanning electron microscope.

The results are shown below in Figure 1 and Figure 2, and Table 3 and Table 4. In individuals exposed to wood smoke, the valves have twice as much mineralized area (*p* = 0.031) and 2.7 times the presence of minerals (*p* = 0.041). Calcium (*p* = 0.031) and phosphorus (*p* = 0.022) are the most abundant minerals. Additionally, the lower amount of sulfur (*p* = 0.031) in exposed individuals suggests that calcification occurs more in the form of phosphates than sulfates, compared to those not exposed. On the other hand, iron shows a forty-six-fold decrease (*p* = 0.032) in individuals exposed to smoke compared to those not exposed.

### 3.2. Mineralization Analysis of Microvesicles

Individuals exposed to wood smoke have a greater amount of calcium and phosphorus in their valves. These data and the MV images are shown with the intention of highlighting that the MVs actively participate in the valve mineralization process by transporting minerals on their surface.

## 4. Discussion

Currently, the study of multiple chronic diseases integrates genetic, epigenetic, and environmental determinants to understand the mechanisms that regulate their development and progression [14]. In particular, environmental factors have been shown to have consequences for the development of different pathological conditions [6]. Although the underlying mechanisms remain largely unknown, particularly in humans, mechanistic insights may emerge from observational and experimental ex vivo human studies. In this study, we demonstrate the association of chronic wood smoke exposure with structural damage and mineralization of aortic valve tissue using imaging-based evidence.

The results show that the main elements, size of the lesion, and the percentage of mineralization in the aortic valves are more extensive in individuals exposed to wood smoke. Environmental pollution, the combustion of different materials such as tobacco, the smoke produced in wildfires, and in other ways, allow for exposure to ketones, carboxylates, and phenolates that behave as chelating agents with transition metals, particularly iron (Fe) [15], altering the cellular homeostasis [16]. These chelating structures generate reactive oxygen species (ROS) that, in the lungs of animal models, induce phagocytosis and iron accumulation, increase inflammatory activity, and promote lung injury [17].

In our study, the greatest disorders occurred in male patients with more than 30 years of exposure to wood smoke, which corresponds to a group of patients in which the majority are farmers and are thus probably exposed in the practice of burning the land before cultivating their plots in the countryside. While we propose that wood smoke exposure may contribute to valve disease development, it is crucial to recognize that the typical onset of degenerative aortic valve disease occurs at an advanced age, together with accumulation of traditional atherosclerotic risk factors such as hypertension, abnormal lipid profile, and related biomarkers such as Endothelin 1 (ET-1) [18,19]. Our results did not exactly align with the profile commonly seen in degenerative aortic valve disease. In our patient’s population, aortic valve disease occurred earlier in exposed persons, a fact probably related to their better lipid profile, thus supporting the hypothesis that wood smoke-related mineralization might play a distinct role in valve disease, contrasting with the profiles commonly seen in degenerative aortic valve disease.

The microenvironment has been established as a factor that has an impact on the modulation of the content of MVs and, therefore, its biological activity and interaction with distinct cellular targets through the transport of different molecules, such as growth factors, lipids, or miRNAS, mainly [20].

Regarding the concentration of MVs, we observed a lower concentration in patients exposed to wood smoke. Of note, MVs were quantified in the systemic circulation, a condition that does not necessarily reflect the valve microenvironment. We want to highlight that, even in this circumstance, the image of the MVs with the minerals is strong evidence that proves the concept. For confirming causality, a longitudinal study could be necessary.

In previous publications, with the SEM–EDS technique, we described the calcification of the coronary artery in patients with ischemic coronary disease [21], in children with congenital malformations [22], and the image of a crystalline structure in an aortic valve [5]. Despite being a highly precise technique that allows for the detection of the structure and composition of tissues as well as the quantification of minerals present in the valves, SEM–EDS is an ex vivo technique, not useful for diagnostic or clinical monitoring purposes. Therefore, the knowledge of the interaction between epigenetic, environmental, and visual support factors such as SEM–EDS will allow for the establishment of new strategies to detect structural changes in the pathophysiology associated with mineralization processes in the aortic valve [23].

## 5. Conclusions

The ex vivo technique does not allow for a direct estimation of the functionality of the valves in patients, but the information it provides allows for us to corroborate, in a limited group of patients, the exposure to PM2.5 due to environmental pollution. Combustion of different materials, such as tobacco and smoke produced in forest fires, increased the mineralization of the aortic valve, and the images obtained from MVs show their epigenetic participation.

Our study uniquely demonstrates imaging-based evidence of structural damage and mineralization in aortic valve tissue, with chronic wood smoke exposure emerging as a potential causative factor. Comparative analyses between exposed and unexposed individuals highlighted these structural differences, suggesting that MVs bearing minerals may play a mediating role. These findings provide objective evidence of valve pathology, extending beyond the subjective symptoms commonly associated with this environmental hazard.

## Figures and Tables

**Table 1:** Demographic characteristics of the patients, segmented according to whether they were exposed to wood smoke.

Variables	Exposed (*n* = 11)	Not Exposed (*n* = 54)
Age (years)	63.0 (56.0–67.0)	58.0 (34.8–65.5)
Sex *n* (%)	Women: 3 (27.3) Men: 8 (72.7)	Women: 25 (46.3) Men: 29 (53.7)
Body mass index (kg/m^2^)	24.5 (23.5–27.3)	26.0 (23.4–28.2)

Variables are shown as the median (interquartile range) or *n* (%) due to the type of data and its distribution. Shapiro–Wilk test, Mann–Whitney U test or chi-square test with the SPSS v23.0 program. Mean values were not statistically significant (*p* > 0.05). % = percentage, kg/m^2^ = kilogram/square meter.

**Table 2:** Laboratory studies presented according to exposure or not to wood smoke.

Variables	Exposed (*n* = 11)		Not Exposed (*n* = 54)	
Hematic biometry:				
Hemoglobin (g/L)	9.8 (8.4–11.3) *		12.7 (11.4–14.1)	
Hematocrit (%)	29.2 (24.7–34.1) *		38.6 (34.2–42.4)	
Platelets (×10^3^/µL)	201.5 (140.0–245.0)		178.0 (150.8–215.3)	
MCV (fL)	88.4 (84.8–91.4)		90.0 (86.9–93.1)	
MCH (pg)	30.5 (29.0–31.4)		30.0 (29.1–31.5)	
MPV (fL)	10.0 (8.6–10.7)		9.3 (8.5–10.0)	
Lipids:				
Total cholesterol (mg/dL)	120.1 (92.9–217.6)		148.9 (118.2–177.2)	
LDL (mg/dL)	66.9 (45.6–154.8)		94.1 (68.6–116.5)	
HDL (mg/dL)	49.5 (35.9–54.0)		36.9 (31.1–44.6)	
Triglycerids (mg/dL)	100.2 (79.3–123.5)		114.0 (87.7–163.9)	
Blood chemistry:				
Glucose (mg/dL)	97.0 (90.3–106.9)		109.6 (91.3–156.0)	
Creatinine (mg/dL)	0.850 (0.683–1.065)		0.915 (0.750–1.103)	
Urea (mg/dL)	17.8 (14.3–21.0)		17.7 (12.8–22.6)	
Serum Calcium (mg/dL)	8.9 (8.6–9.3)		9.0 (8.6–9.7)	
Serum Chlorine (mM/L)	103.9 (100.6–104.6)		102.1 (99.1–104.4)	
Serum Phosphorus (mM/L)	3.8 (3.1–4.3)		4.0 (3.6–4.4)	
Serum Sodium (mM/L)	136.0 (135.0–139.2)		138.0 (135.8–140.2)	
Serum Potassium (mM/L)	4.2 (4.1–4.4)		4.2 (3.9–4.5)	
Other determinations:				
Lp(a) (mg/dL)	<65 years	>65 years	<65 years	>65 years
	3.7 (2.6–5.6)	6.1 (2.3–8.5)	3.8 (2.3–10.7)	5 (2.7–13.1)
MVs (mg/mL)	130.2 (97.0–163.5)		161.0 (99.7–206.5)	

HDL = high-density lipoprotein, LDL = low-density lipoprotein, MCH = mean corpuscular hemoglobin, MCV = mean corpuscular volume, and MPV = mean platelet volume. Lp(a) = lipoprotein (a), MVs = microvesicles, g/L = gram/liter, % = percentage, µL = microliter, fl = femtoliter, pg = picogram, mg/dL = milligram/deciliter, mM/L = millimolar/liter. All variables are shown as the median (interquartile range). * *p* < 0.05 was considered statistically significant.

**Table 3:** Mineralized area, optical density, and elements present in the aortic valves of the patients; data sectioned according to exposure or not to wood smoke.

VARIABLES	EXPOSED (*n* = 11)	NOT EXPOSED (*n* = 54)
Mineralized area (µm^2^)	835.3 (432.1–1711.9) *	407.5 (216.0–901.9)
OD (px × 10^3^)	84.5 (31.7–184.8) *	30.6 (18.5–77.0)
Elements (%)		
Calcium (Ca)	52.59 (28.13–54.29)	34.99 (22.50–52.86)
Sulfur (S)	1.17 (0.97–2.06) *	6.39 (1.25–22.85)
Phosphorus (P)	32.18 (14.20–40.14) *	10.18 (2.51–20.80)
Silicon (Si)	0.22 (0.06–0.50)	0.56 (0.17–3.29)
Iron (Fe)	0.94 (0.34–7.46) *	10.22 (0.98–26.44)
Sodium (Na)	1.79 (0.69–2.89) *	0.78 (0.35–1.67)
Magnesium (Mg)	0.39 (0.33–0.77)	0.31 (0.16–0.55)
Aluminum (Al)	0.32 (0.10–0.48)	0.21 (0.08–0.67)
Potassium (K)	0.23 (0.12–0.46)	0.37 (0.12–1.21)
Titanium (Ti)	0.22 (0.08–0.34) *	0.54 (0.17–0.96)
Chromium (Cr)	0.07 (0.05–0.69) *	0.68 (0.13–2.79)
Manganese (Mn)	0.62 (0.18–1.13)	0.66 (0.30–1.21)

All variables are shown as the median (interquartile range) due to the type of data and its distribution. * *p* < 0.05 was considered statistically significant. Shapiro–Wilks and Mann–Whitney U tests were performed with the SPSS v23.0 program. OD = optical density, µm^2^ = micrometers square, px = pixel, % = percentage.

**Table 4:** Mineral elements present in the microvesicles of three patients undergoing valve replacement.

Elements	Minerals (%)
Calcium (Ca)	0.69 ± 0.68
Sulfur (S)	1.00 ± 0.50
Phosphorus (P)	0.14 ± 0.03
Silicon (Si)	9.21 ± 9.09
Iron (Fe)	0.37 ± 0.37
Sodium (Na)	5.76 ± 3.24
Magnesium (Mg)	0.05 ± 0.05
Aluminum (Al)	0.08 ± 0.02

All variables are shown as averages ± SD. % = percentage, SD = standard deviation.

## Data Availability

Further information can be directed to the corresponding authors.
